# Clinical use of cone-beam computed tomography in Western Norway: a referral-based retrospective study

**DOI:** 10.2340/aos.v83.41943

**Published:** 2024-09-26

**Authors:** Marianne L. Vollan, Linda Cecilie Kleppe Hasselgren, Xie-Qi Shi, Malin V. Jonsson

**Affiliations:** a Oral and Maxillofacial Radiology, Department of Clinical Dentistry, University of Bergen, Bergen, Norway; b Oral Health Centre of Expertise in Western Norway (TkVestland), Bergen, Norway; c Faculty of Odontology, Malmö University, Malmö, Sweden

**Keywords:** CBCT, clinical indication, referral, justification

## Abstract

**Objective:**

To elucidate cone-beam computed tomography (CBCT) referral profiles in Western Norway.

**Materials and methods:**

In all, 3,031 referrals to oral- and maxillofacial radiologist were reviewed. Patient data were retrieved retrospectively from electronic charts. The patient’s age, gender, and perceived clinical indication were noted, as well as relevant medical and dental history and whether the referring clinician was a general dentist or held a clinical dental specialty.

**Results:**

A total of 2,680 referrals fulfilled the inclusion criteria (UiB *n* = 1,471, and TkVestland, *n* = 1,209). The female:male ratio was 1,427:1,253. Mean age was 33 years – 35 years for females compared to 31 years for males (*p* < 0.001).

The most common clinical indications were related to impacted teeth (29%), endodontic issues (17%), cleft lip palate (12%), and resorptions (10%). Less common were bone lesions, implant planning, trauma to the teeth or jaws, atypical orofacial pain, and temporo-mandibular joint (TMJ). The patient age-profiles mirrored differences in indications within the cohort. Most referrals were from specialist dentists such as orthodontists, oral surgeons, and endodontists. Interestingly, 543/2,680 (20%) referrals were from general dentists.

**Conclusions:**

Specialist dentists such as orthodontists, oral surgeons, and endodontists refer most patients for clinical indications such as impacted teeth, endodontic issues, and resorptions.

## Introduction

Despite guidelines on good clinical practice concerning diagnostic use of radiation [1, 2] and growing scientific evidence regarding the efficacy of cone-beam computed tomography (CBCT), there is still a wide discrepancy among dental professionals with regard to when a referral to CBCT is indicated. Misuse of radiation in frequency and unnecessary high exposure exists, such as using CBCT imaging as a screening method for orthodontic patients [3]. The requirements for performing CBCT examinations differ between nations [4–6]. In Norway, legal requirements of CBCT equipment entail registration of the machine as well as documentation of staff formal competence. Other confounding factors for the discrepancy of CBCT use may be because of varied awareness of stochastic radiation risk, legal implications, and insufficient clinical evidence on the efficacy of CBCT [2, 7, 8].

The total number of registered CBCT machines has increased immensely in the past decade, and at the beginning of 2023, there were 175 machines registered at the Norwegian Radiation and Nuclear Safety Authority (*personal communication*, https://dsa.no, 2023). In Sweden, there were 343 registered CBCT machines at the Swedish Radiation Safety Authority (*personal communication*, www.stralsakerhetsmyndigheten.no, 2023). Hence, the number of machines per inhabitant is around 3.2 per 100.000 inhabitants in the two neighbouring countries; 343 CBCT machines/10.582.000 registered persons in Sweden, and 175 CBCT machines/5.504.000 persons registered in Norway, in the first quarter of 2023.

In a Swedish study from 2019, approximately 8% of dentists in Sweden reported to have access to CBCT, and in 75% cases the dental nurses perform the CBCT image acquisition. In 56% of the clinics having access to CBCT, more than 75 CBCT examinations were performed yearly. Access to CBCT was associated with dentists’ work in the public dental health service or in a group practice, and whether the dentist had undergone some kind of postgraduate course in oral radiology [9]. In contrast, a questionnaire-based study from Norway, published in 2015, reported that clinical dental specialties owning their own CBCT in Norway commonly were periodontists and oral and maxillofacial surgeons. The most common indications for CBCT were related to treatment planning [10].

A survey performed among the United Kingdom (UK) dental practices, published in 2019, revealed a wide range of CBCT equipment in use, although the reported number of scans was low. One third of respondents had acquired their CBCT machine within the last year. Most clinical use was related to implant dentistry in adult patients, and small or medium field of view (FOV) scans were the most used. Less than 20% of respondents could provide detailed exposure parameters. The authors concluded there was no evidence of excessive CBCT use in the UK dental practices; the typical was small FOV scans for implant dentistry. Only 8.4% of the practices performed examinations on children and young people [5].

During 2016–2018, around 30% of the Norwegian population were entitled to free or subsidised treatment in the Norwegian public dental health care system; the vast majority (2/3) of these individuals were 18 years or younger [11]. Depending on which category the patient belongs to, they pay 0–25% of the total treatment cost. For the remaining 70% of the public, the patient must cover the treatment costs by themselves. However, for this latter group of patients, the National Insurance Scheme covers part of the cost for some named conditions, arranged in 15 allowance categories [12]. If the clinical indication does not fit with one of the defined categories, the patient must cover the entire cost.

**Figure 1:**
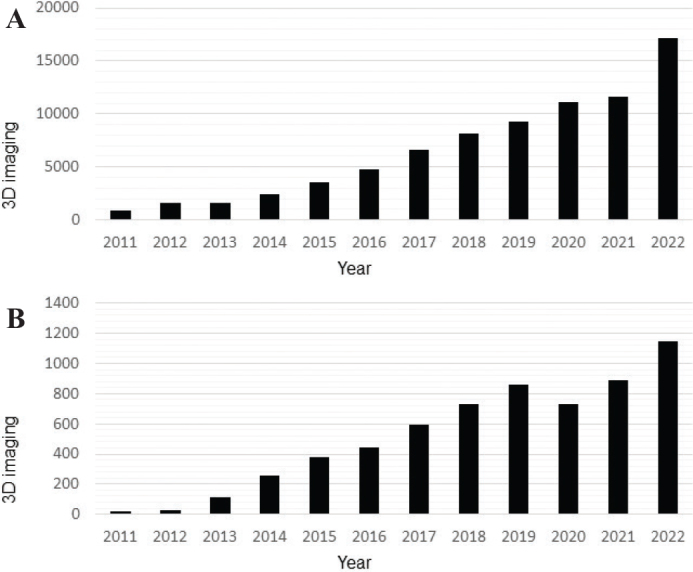
(A) 3D imaging with financial support from social security (HELFO) in Norway 2011–2022. The number of examinations using 3D imaging in Norway has steadily increased in the past 12 years, here illustrated by the number of volumes obtained when the patients are entitled to some kind of support from social security (HELFO). (B) 3D imaging with financial support from social security (HELFO) in Vestland county 2011–2022. Information obtained from the Norwegian Health Economics Administration (HELFO). In addition to the numbers shown in the figure are CBCT examinations performed without support from HELFO at private dental clinics, hospital clinics, as well as the University (UiB) dental clinic and the public oral health care center of expertise in Bergen (TkVestland) clinic.

The number of CBCT examinations performed with social security financial support from the Norwegian Health Economics Administration (HELFO) (*personal communication*, www.helfo.no, 2023), has increased steadily in the last 12 years, in Norway as a whole (Figure 1A) and in the western part of Norway (Vestland county) (Figure 1B). Although not representative for the total number of acquired CBCT examinations per year, they indicate a general level. In view of the increasing interest in applications and use of CBCT, the aim of this study was to elucidate and verify the CBCT referral profiles of two large dental specialist clinics in Vestland county, Norway. The overall aim was to reflect over and quality assure clinical routines in the justification process of CBCT referral.

## Materials and methods

### Patient cohort

Study material was retrospectively obtained from patients’ electronic data charts (Opus Dental version 7.1, Opus systemer AS, Hvalstad, Norway), with a focus on referrals to and radiology reports from two dental clinics, namely the University Dental Clinic, University of Bergen (UiB), and the Public Oral Health Centre of Expertise Clinic (TkVestland), both located in Bergen, Vestland County, Norway. As a clinical routine at both the UiB and TkVestland dental clinics, all referrals for CBCT examination had previously been individually assessed by an oral- and maxillofacial radiologist or clinical fellow in oral- and maxillofacial radiology, to evaluate justification and optimisation according to local, national, and international guidelines [2, 13–15]. From the TkVestland, referrals (*n* = 1,420) dating from 2013 to 2019 were available. From the UiB, referrals (*n* = 1,611) dating from 2016 to 2018 were readily available. In total, 3,031 referrals were available for review; 2,117 of these were from 2016 to 2018.

From the referrals, information regarding the patient’s age and gender, along with relevant medical and dental history was noted. The formulated clinical indication, the planned treatment (when stated), as well as region – tooth/teeth (region/quadrant), or jaw (maxilla/mandible) were also noted. In some referrals, several clinical questions and regions were addressed in the same referral, but for convenience only the main question and region was noted and included in the further analyses, resulting in one clinical question per referral. With regards to the referring dentist’s occupation, they were classified as general dentist or specialist in oral surgery, endodontics, orthodontics, paediatric dentistry, periodontology, prosthodontics, or specialist in cariology (preventive/restorative dentistry). In some cases, referrals were from clinical fellows undergoing dental specialist training; if so, the referring dentist was classified as specialist in the clinical specialty in question. Some of the fellows also referred patients from private, general practice; if so, the referring dentist was classified as a general dentist.

### Ethical approval

The study was designed as a quality assurance project and reported to the Norwegian Centre for Research Data – NSD; for TkVestland project number 60564 and UiB project number 51391. Being a quality-assurance study, informed consent from the individual patient could be waivered. All measures were taken to keep patients and referring dentists/clinics anonymous in the analyses; only strictly necessary information was transferred to the database. Names and other identifying factors were kept separate from the data analyses.

### Inclusion and exclusion criteria

For referrals to be included in all analyses, a written radiological report describing CBCT acquisition and radiological findings was set as a criterion.

In certain cases, referrals and reports indicated that the patients were part of research projects following specific protocols such as several exposures over several years, and repeated referrals were later excluded from the analyses. Similarly, patients belonging to a collaborative research project encompassing the two clinics (UiB and TkVestland) on juvenile idiopathic arthritis (JIA) were also excluded from the UiB cohort to avoid overlap in data. Referrals that for some reason did not result in CBCT acquisition were also excluded, as were referrals for sialography and soft tissue calcifications, when the clinical indication had not justified CBCT examination (Table 1).

**Table 1:** Referrals not resulting in CBCT acquisition at TkVestland (n = 102) and UiB (n = 16) and excluded from further analyses.

	TkVestland	UiB
**CBCT examination not justified**	**28**	**16**
Based on presentation of clinical problem	13	16
Sufficient with panoramic radiograph, CBCT not justified	3	
Sufficient with intraoral images and/or clinical examination	9	
Need of clinical examination	3	
**Second opinion/consultation**	**15**	
CBCT examination justified but not acquired*	**59**	
No-show/misunderstood appointment	19	
Cancelled/postponed appointment	19	
Other*	21	
**Total**	**102**	**16**

CBCT: cone-beam computed tomography.

* CBCT machine not available or not possible to perform acquisition (*n* = 8), patient belongs to another clinic (*n* = 5), waiting for appointment (*n* = 8).

### Justification process

All referrals had previously been individually evaluated by the staff radiologist (TkVestland), and the staff radiologists or senior fellows in oral and maxillofacial radiology (UiB). Justification was based on current European clinical guidelines [2]. Being a retrospective study, calibration exercises were not performed prior to image acquisition and data collection, and the study material therefore represents cross-sectional data based on daily clinical practice in the two clinics in the selected time-period. In a few cases, the clinical problem could not be read from the referral in the digital chart, but if a CBCT had been acquired, it was assumed that the clinical problem adhered to justification guidelines and the patients were kept in the cohort.

### Statistical analyses

Descriptive analyses of patient characteristics in terms of age, gender, clinical indications, and professional status of referral dentists, were performed using Statistical Package for the Social Sciences (SPSS) (version 27.0.1.0). T-test was used to compare the mean age between patients allocated at either UiB or TkVestland. To investigate correlations between categorical variables, the Pearson chi-square test of independence was used. *P*-values < 0.05 were regarded as statistically significant.

## Results

### Patient cohort

**Figure 2:**
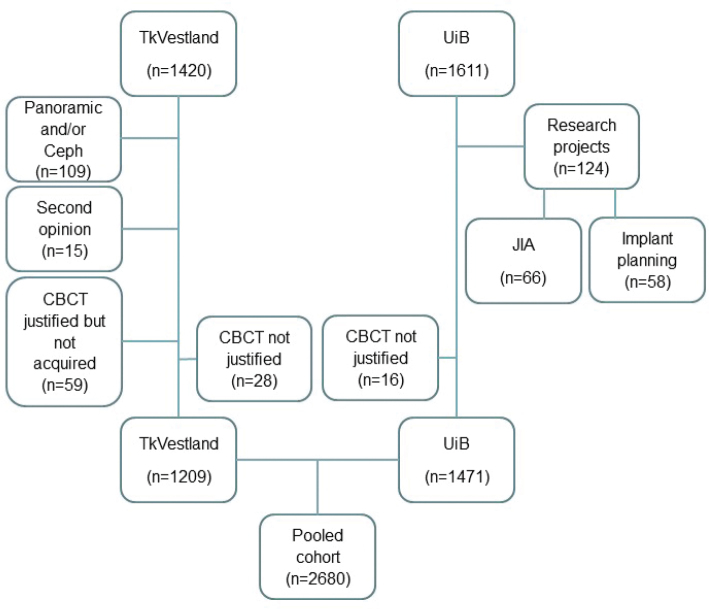
Inclusion and exclusion criteria. For referrals to be included in all analyses, a written radiological report confirming CBCT acquisition was termed mandatory. Referrals resulting in multiple CBCT acquisitions as part of research projects were excluded, as were cases where overlap between cohorts was possible. Referrals with clinical questions not justifying CBCT examination were also excluded. Cone-beam computed tomography (CBCT), Cephalogram (Ceph), Juvenile idiopathic arthritis (JIA), Oral health center of expertise in Western Norway (TkVestland), University of Bergen (UiB).

In total, 1,420 written referrals to the staff oral and maxillofacial radiologist at TkVestland were located. After reading all referrals and available radiology reports (*n* = 1,332), the material consisted of a group of patients where panoramic and/or Cephalogram (Ceph) images (*n* = 109), CBCT alone (*n* = 1,191), or CBCT and panoramic (*n* = 18) images had been acquired. Among these, 1,209 referrals fulfilled the inclusion and exclusion criteria, and were included in the further analyses (Table 1, Figure 2).

At the UiB dental clinic, a patient cohort consisting of 1,611 written referrals was available. In total, 1,471 referrals to the UiB dental clinic fulfilled the inclusion and exclusion criteria, and were included in the further analyses (Figure 2).

### Cone-beam computed tomography patient characteristics

Referrals for CBCT to the public dental specialist clinic (TkVestland), that ended up with acquisition of a CBCT or a CBCT in addition to a panoramic radiograph, were included in the further analyses (*n* = 1,209), in addition to patients referred to the UiB dental clinic for CBCT examination (*n* = 1,471) (Table 2). The male patients were younger than the female patients in both the cohorts, but the difference was only significant for the UiB dental clinic patients (*p* = 0.021) (Table 2).

**Table 2:** Patient characteristics.

	UiB (*n* = 1,471)	TkVestland (*n* = 1,209)	*p*
**Mean age** (SD)	44.2 (20.7)	20.1 (16.5)	< 0.001
Females	45.2 (20.1) (*n* = 856)	20.8 (17.2) (*n* = 571)	< 0.001
Males	42.7 (21.4) (*n* = 615)	19.5 (15.9) (*n* = 638)	< 0.001
**Min-max**	6–95	4–86	
**Mode age**	16 (*n* = 42)	16 (*n* = 164)	
**Median age**	45	15	

SD: standard deviation.

Pooling the cohorts (*n* = 2,680), mean age (standard deviation [SD]) was 33.3 (22.4) years, with the youngest patient 4 years old and the oldest 95 years old, median age 25 years, and mode age 16 years. The TkVestland patients were younger than the UiB patients (*p* < 0.001). Mean age in the female part of the pooled cohort (*n* = 1,427) was 35.4 (22.4) years compared to 30.9 (22.1) years for the males (*n* = 1,253), a statistically significant difference (*p* < 0.001).

**Figure 3:**
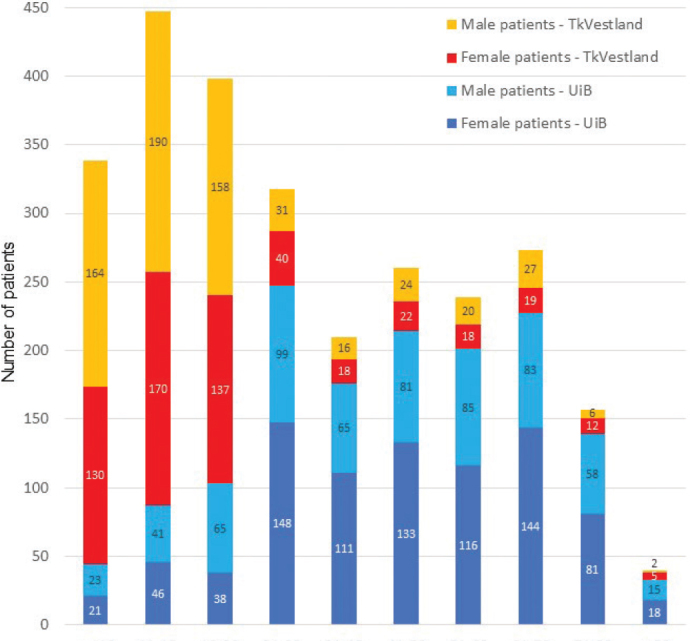
Age intervals and gender distribution in the cohort. The UiB and TkVestland subgroups illustrate differences in age-profiles and gender-profiles.

In the pooled cohort, female:male ratio was 1,427:1,253 (1.1). Interestingly, for the public dental specialist clinic (TkVestland) the ratio was reversed, with a female:male ratio of 571:638 (0.89) whereas the UiB dental clinic had a higher female:male ratio of 856:615 (1.4). The difference was statistically significant (*p* = 0.019). The age and gender distribution of the two sub-cohorts is illustrated in Figure 3.

### Clinical indications

**Figure 4:**
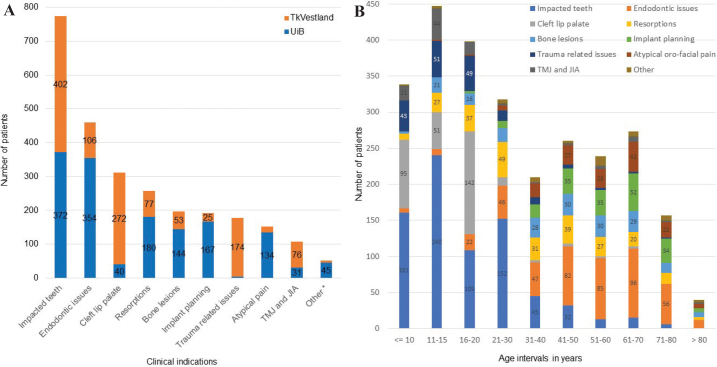
(A) Clinical indications in referrals to CBCT to UiB and TkVestland. Numbers lower than 20 not shown. (B) Differences in clinical indications characterised by age intervals. Numbers lower than 20 not shown.

In total, 2,739/3,031 (90%) of the referrals had clinical indications that justified CBCT image acquisition. The overall most common indications when considering all CBCT referrals (*n* = 2,680) were related to impacted teeth (*n* = 774; 29%), endodontic issues (*n* = 460; 17%), cleft lip palate (*n* = 312; 12%), and resorptions (*n* = 257; 10%) (Figure 4A). In total, these four groups made up two thirds of the referrals.

Looking at the separate clinics, resorptions, implant planning, atypical pain issues, and bone lesions were the most common clinical indications at UiB, whereas TkVestland had a large number of trauma-related issues, resorptions, and TMJ/JIA. The patient age profiles differed both when comparing the two clinics (Figure 3) as well as when comparing the various clinical indications (Figure 4B). The gender ratios were also different when comparing clinical indications in the UiB and TkVestland sub-cohorts (Table 3), with more female patients overall, except for the cleft lip palate, and the trauma-related issues. For atypical orofacial pain issues, endodontic issues and TMJ/JIA, the female predominance was especially pronounced.

**Table 3:** Gender ratios in relation to clinical indications in the pooled cohort and sub-cohorts.

Gender (women/men)					
Referral question	*n* (total)	All	UiB	TkVestland	*p*
Impacted teeth	774	432 / 342	212 / 160	220 / 182	0.526
Endodontic issues	460	280 / 180	227 / 127	53 / 53	0.009
Cleft lip palate	312	94 / 218	11 / 29	83 / 189	0.698
Resorptions (all)	257	132 / 125	90 / 90	42 / 35	0.504
Bone lesions	197	108 / 89	81 / 63	27 / 26	0.507
Implant planning	192	105 / 87	95 / 72	10 / 15	0.114
Trauma	178	83 / 95	1 / 3	82 / 92	0.380
Atypical orofacial pain	152	105 / 47	90 / 44	15 / 3	0.163
TMJ incl. JIA	107	59 / 48	25 / 6	34 / 42	< 0.001
Other*	51	29 / 22	24 / 21	5 / 1	0.163
The whole cohort	2,680	1,427 / 1,253	856 / 615	571 / 638	< 0.001

TMJ: temporo-mandibular joint, JIA: juvenile idiopathic arthritis.

* Clinical issues with 15 or fewer referrals.

Within the issues related to impacted teeth, third molars were the most common retained teeth in referrals at UiB, and canines at TkVestland; molars were the most common teeth with endodontic issues at UiB, and incisors at TkVestland, and resorptions were examined in molars, incisors and premolars, at both clinics.

### Referring dentists

**Figure 5:**
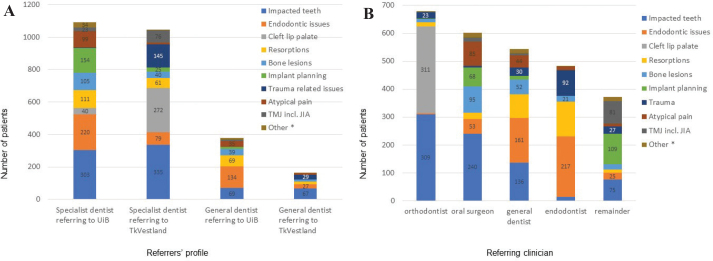
(A) Clinical indications in referrals in the sub-cohorts, from specialist dentists and general dentists. Numbers lower than 20 not show. (B) Clinical indications in referrals in the pooled cohort, from various specialist dentists and general dentists. The remainder group contains prosthodontists, paediatric dentists, periodontists, medical doctors, and cariologists. Clinical indications regarding sinus, periodontal problems, infection/inflammation, controls, implant-related issues not related to implant planning, and clinical issues not clearly stated were grouped in ‘others’ because of low numbers in each category. Numbers lower than 20 not shown.

Most referrals (80%) in the pooled cohort were from specialist dentists; the proportion of referrals from general dentists was higher at UiB, 26% compared to 14% at TkVestland (Figure 5A). The main clinical indications differed when comparing specialist dentists and general dentists. Apart from impacted teeth, specialist dentists referred endodontic issues and implant planning to the UiB clinic, and cleft lip palate issues and trauma-related issues to TkVestland. To the UiB clinic, general dentists referred endodontic issues, impacted teeth and resorptions, whereas impacted teeth, trauma related issues and endodontic issues were most frequently referred by general dentists to the TkVestland clinic.

In the pooled cohort, most referrals were from dental specialists such as orthodontists (25%), oral surgeons (22%) and endodontists (18%), as well as general dentists (20%). The remaining were prosthodontists (5%), paediatric dentists (3%), periodontists (3%), and medical doctors (3%). The distributions of clinical indications for each category are presented in the bar chart shown in Figure 5B. In the ‘remainder’ group, referrals regarding implant planning were from prosthodontists and periodontists, TMJ/JIA from MDs as part of a research project, and impacted teeth were referred from paediatric dentists.

## Discussion

In this retrospective study, cross-sectional results based on referrals/reports at the two largest dental clinics in Vestland county, Norway, the TkVestland clinic, and the UiB are reported. The patients that come to these two clinics are representative of the general adult population, mainly UiB, as well as politically prioritised groups, mainly TkVestland, and the patients’ characteristics and concerned clinical indications differ accordingly. The absolute numbers of CBCT acquisitions at UiB and TkVestland counting CBCT examinations both with and without support from social security/HELFO, have increased from around 200 in 2013 to more than 1.000 in 2019; these acquisitions are part of the HELFO-supported examinations, supporting the assumption that UiB and TkVestland together indeed represent the largest specialist clinics in Vestland county, Norway, with regard to CBCT referrals and examinations. In perspective, the number of inhabitants in Vestland county (before 2020: Hordaland county, and Sogn and Fjordane county) increased from 626.027 in 2016, to 630.229 in 2017, and 632.729 in 2018 [16], whereas the number of CBCT examinations performed at UiB and TkVestland in these years increased from 621 in 2016 (99/100.000) to 702 in 2017 (111/100.000), and 794 in 2018 (125/100.000).

In both the cohorts, issues related to impacted teeth were the most common clinical issue in the referrals. The TkVestland clinic consists of children, adolescents, and adults with either age- or health-related circumstances privileging public dental care. For several years, the TkVestland dental clinic has served as a centralised treatment clinic for children and adolescents with cleft lip palate malformations in western Norway. Furthermore, the TkVestland cohort contained a high number of patients with JIA, as a result of a research collaboration. Taking this into consideration, the high number of patients referred to CBCT examinations in these otherwise rare groups of patients, as well as for the accumulation of CBCT examinations for 16-year-olds, was accounted for. Taking out the cleft lip palate and TMJ/JIA related issues from the TkVestland cohort, the impacted teeth were mostly canines, and the other common clinical issues were dental/orofacial trauma related issues, endodontic issues, and resorptions. Impacted third molars, endodontic issues, root resorptions, and implant planning were common clinical issues in the older, UiB part of the cohort, in addition to bone lesions such as suspected cysts, benign tumours or radiopaque/sclerotic lesions, and issues related to atypical orofacial pain. In the UiB part of the cohort, the number of referrals to implant planning in an ongoing research project involving partly edentulous adults was corrected for multiple referrals of the same clinical indication.

Overall, most referrals were from orthodontists, oral surgeons, and endodontists; orthodontists because of the high number of cleft lip palate issues. The general dentists contributed 20% of referrals, concerning endodontic issues, impacted teeth, and root resorptions. The proportion of referrals from general dentists was almost double in the UiB cohort compared to the TkVestland cohort, a consequence of the main sources of patients and clinical issues because of public policy.

Several questionnaire studies have reported the profile of CBCT use in the UK [5, 17], Scandinavian countries [4, 10], Turkey [18, 19], and Japan [20]. The results from these surveys reflected dentists’ knowledge and standpoint in the use of CBCT, which can be compared with the current recommendations of CBCT applications [2]. In previous studies, mainly from private dental clinics, most clinical uses in adults are related to implant dentistry and endodontic diagnostics in adult patients; small or medium FOV scans are most common [4, 5, 10]. These findings coincide with the adult part of the current cohort; implant planning made up 7% of the clinical issues in all referrals, with 99% of the patients older than 18 years. In the 17% that made up referrals for endodontic issues, 95% of the patients were older than 18 years.

Localisation of impacted teeth and disorders in tooth eruption were the most common reasons for CBCT examination [10, 17, 20], making up 29% of the referrals in the current cohort, with 61% of the patients aged 18 years or younger, and 76% of these belonging to the public specialist clinic (TkVestland). In total, 40% of the referrals in the pooled cohort were for patients 18 years or younger; 81% of them belonged to the TkVestland part of the cohort, where most patients are younger because of policy. In the UK, Yalda et al. found only 8.4% of practices performed examinations on children and young people and concluded that there was no evidence of excessive CBCT use in UK dental practices [5]. However, looking only at the university clinic (UiB), less than 14% of the patients are 18 years or younger, with 58% of the referrals regarding impacted teeth, close to 18% regarding cleft lip palate issues, and almost 12% regarding root resorptions. In comparison to surveys concerning the use of CBCT based on questionnaires with 40–70% response rates [4, 9, 10] which are more likely subjective and respondent dependent, the current cohort represents cross-sectional data collected from the two largest dental clinics in Vestland county, Norway. More like the current study design, a retrospective study conducted at three dental hospitals in the UK, examining the use of CBCT in paediatric patients revealed that CBCT examinations performed on patients under the age of 18 constituted 13.7% of all scanned patients. The CBCT was used more frequently in the >13 year age group, the most common clinical use was the localisation of unerupted teeth in the anterior maxilla and the detection of root resorption [21].

According to national legislation in Norway and Sweden, the justification process for CBCT examination should be performed by oral- and maxillofacial radiologists. In the present study, a very low number of referrals had been considered not justified; only 2% at TkVestland and 1% at the UiB clinic, based on referral content. Being a retrospective study though, it is possible that the numbers are biased. In addition, both clinics work very closely with several of the clinicians who regularly refer patients, enabling a discussion of the clinical indication and justification prior to receiving the referral. Similarly, discussions regarding the clinical task may also take place after the referral is received.

The efficiency of CBCT, as pointed out many times earlier, should be analysed at a higher grade of the efficacy ladder of a diagnostic modality. When writing a referral for CBCT examination, the required diagnostic information is commonly specified, ensuring the CBCT indication on diagnostic accuracy level. The CBCT is superior when used to detect root resorptions in comparison to 2D images [22–24]. However, there is no consensus on whether increased precision of root resorption detection plays any major role in altering or affecting therapeutic thinking [23, 25–29]. Whether the acquired 3D information affects therapeutic thinking has only been sparsely investigated. A recent study from Hermann et al. on the therapeutic efficacy level, found that CBCT examination of maxillary third molars changed the treatment plan in 32% of the cases [30]. Ihlis et al. found that when CBCT indication was performed on the therapeutic thinking level, 50% of the acquired CBCTs of impacted canines were not justified [29]. This finding emphasised the importance of referral content and interdisciplinary therapy planning. By providing information about the planned therapy and thus the reasoning of CBCT, it is possible to further improve the benefit-risk assessment of CBCT examinations.

Numerous clinical studies have provided evidence for the use of CBCT on the diagnostic accuracy level for various clinical indications when compared to conventional 2D dental radiographs [29, 31–36]. The contribution of ionising radiation from a diagnostic tool in patient management process shall preferably be based on higher levels of the efficacy ladder [37]. Increases in efficacy at lower levels (e.g. technical or diagnostic accuracy) will not guarantee commensurate improvement at higher levels (e.g. therapeutic and treatment outcome). A significant lack of clinical studies of CBCT on therapeutic efficacy and treatment outcome has resulted in low-grade evidence on the benefit of CBCT. Consequently, uncertainty in the context of justification may be expected, particularly concerning paediatric applications [17, 21, 38].

The current retrospective study has several strengths; it provides objective, cross-sectional data reflecting daily, clinical use of CBCT in a significant part of Vestland county, Norway. The cohort contains a considerable number of cases compared to earlier studies. Limitations include the retrospective study design, where it was not possible to standardise the justification process in terms of indication level, access to 2D radiographs exposed at referred dental clinics, and referral quality. Also, only one clinical indication was noted per referral, possibly influencing the absolute numbers of clinical issues and limiting information with regards to commonly associated clinical questions.

In conclusion, the majority of the CBCT referrals were from specialist dentists such as orthodontists, oral surgeons and endodontists, for clinical indications such as impacted teeth, endodontic issues and resorptions. For most of the referrals, the clinical indication justified CBCT examination. However, with the current retrospective study, the efficacy level of the carried-out CBCT examination was not possible to classify. Further clinical trials are warranted.
